# Musculoskeletal fitness and balance in older individuals (65–85 years) and its association with steps per day: a cross sectional study

**DOI:** 10.1186/s12877-016-0188-3

**Published:** 2016-01-12

**Authors:** H. Lohne-Seiler, E. Kolle, S. A. Anderssen, B. H. Hansen

**Affiliations:** Faculty of Health and Sport Sciences, University of Agder, Service Box 422, N-4604 Kristiansand, Norway; Department of Sport Medicine, Norwegian School of Sport Sciences, Oslo, Norway

**Keywords:** Accelerometer-determined physical activity, Fitness score, Older people

## Abstract

**Background:**

There is limited normative, objective data combining musculoskeletal fitness (MSF), balance and physical activity (PA) among older adults. The aims were therefore to; 1) describe MSF and balance in older Norwegian adults focusing on age- and sex-related differences; 2) investigate the associations among MSF, balance and objectively-assessed PA levels.

**Methods:**

This was part of a national multicenter study. Participants (65–85 years) were randomly selected from the national population registry. We used ActiGraph GT1M accelerometers to measure PA. Balance and MSF were assessed using: one leg standing (OLS), handgrip strength (HG), static back extension (SBE), sit and reach (SR), back scratch right, left arm over (BSR, BSL). Univariate analyses of variance were used to assess sex differences within the different MSF and balance tests and for comparisons among multiple age groups. Linear regression analysis was used to investigate how PA (expressed in 1000 steps increments) was associated with MSF and balance.

**Results:**

85 women and 76 men were included. Mean age (standard deviation (SD)) was 73.2 (5.4) years for women and 72.3 (4.8) years for men. The youngest participants (65–69 years) had significantly better mean OLS- and SBE results compared with older participants. Women (65–85 years) had significantly better mean SR, BSR, BSL and SBE results compared with men (65–85 years). Men had significantly better mean HG results compared with women. No sex differences in mean OLS results were observed. A daily increment of 1000 steps was associated with better mean test scores for OLS- and SBE tests (b = 1.88, 95 % CI: 0.85 to 2.90 (p ≤ 0.001) and b = 4.63, 95 % CI: 1.98 to 7.29 (*p* = 0.001), respectively).

**Conclusion:**

The youngest (65–69 years) had better static balance and muscular endurance in trunk extensors compared with older participants. Older women (65–85 years) had better joint flexibility than older men (65–85 years), whereas older men had better handgrip strength than older women. A higher PA level was associated with better static balance and muscular endurance in trunk extensors in older individuals. This study provides important normative data, and further investigation of trunk endurance and static balance as key foci for PA interventions in elderly is warranted.

## Background

After reaching 30, aging leads to a progressive loss of muscular strength, muscular endurance, joint flexibility [1], and balance [2–4]. Age-induced musculoskeletal fitness (MSF; a comprehensive picture of upper- and lower body muscular strength and muscular endurance, and upper- and lower body joint flexibility) loss may inhibit older people from performing basic functional tasks such as lifting and moving objects, rising from a chair, and walking. MSF is therefore an important determinant of one’s capability to manage daily life activities and maintain functional independence [5–7]. The incidence of falls increases with age; muscle weakness, impaired gait and diminished balance are the most significant risk factors for falling [8, 9]. Fundamentally, fall avoidance challenges the ability to maintain the center of gravity over the base of support whether moving (dynamic balance) or stationary (static balance) [8]. Static balance might therefore be an important component for predicting falls in older adults [10]. Balance-and muscle strengthening activities seem to influence risk factors for falls by increasing muscle strength and balance ability [11, 12]. In turn, such improvements increase one’s ability to remain independent with advancing age [11].

Despite apparent connections between these variables, MSF and balance data collected on apparently healthy elderly, using standardized assessment methods, are scarce [13, 14]. Current knowledge is primarily based on studies that have measured balance [15], or handgrip strength [16–20] separately. Few published studies have focused on an overall fitness evaluation (i.e. a more comprehensive picture of MSF and balance) among older adults [21, 22]. These studies showed that all test scores declined with increasing age. Women scored better on the upper and lower body flexibility tests, whereas men performed better on upper and lower body strength- and balance tests [21, 22]. The majority of the studies mentioned above have all been conducted outside the Nordic countries. In Norway, MSF- and balance data for normative values of individuals 65 years and older have not yet been published.

Physical activity (PA) levels decline significantly with age [23–28]. In older individuals, loss of MSF and balance in combination with decreased PA levels is strongly predictive of falls [29], disability [30], hospitalization [31], reduced quality of life [32], and increased mortality [1, 33]. There are a limited number of studies assessing the associations among MSF level, balance ability and objectively assessed PA levels in older adults. Also, some of the existing studies showed associations [34–37], whereas others did not [8, 38]. It is also somewhat difficult to distinguish which components of MSF (i.e. muscle strength and endurance, and joint flexibility) might be associated with PA level in the studies mentioned above. A study conducted by Aoyagi et al. [38] showed that neither balance nor handgrip strength were related to daily step counts, whereas lower-extremity function (walking speeds and knee extension torque) was positively related to daily step counts in older adults. In contrast, de Melo et al. [34] reported that balance and lower body flexibility were both associated with daily step counts in older adults (mean steps for 3 days: ≥ 6500).

Regular physical activity in older adults is associated with improved functional ability [39], maintained mobility [40], and reduced mortality [41]. Therefore, more knowledge about musculoskeletal fitness- and balance ability in older men and women, and their association with physical activity level, may be of importance towards establishing future preventive health strategies in older adults.

Given these considerations, the aims of the present study were to; 1) describe musculoskeletal fitness and balance in a random national sample of Norwegian older individuals (65–85 years) focusing on age- and sex-related differences, and 2) investigate the associations among musculoskeletal fitness, balance, and objectively-assessed physical activity levels. Based on this the following hypotheses were provided: Among older Norwegian adults the younger individuals have better musculoskeletal fitness and balance ability compared with the older individuals. Older men have better muscle strength and balance compared with older women, whereas older women have better joint flexibility compared with older men. A higher physical activity level is associated with better musculoskeletal fitness and balance ability in older adults.

## Methods

### Design and participants

This study was part of a multicenter study involving 10 test centers throughout Norway [27, 28], and consisted of test phase one (determining physical activity level using accelerometers) and phase two (determining MSF level and balance). A representative sample of 2040 individuals aged 65–85 years, were randomly drawn from the Norwegian population registry. The participants were randomly selected and stratified based on sex, age and geographical place of residence. Study information and informed consent were distributed via mail to the drawn sample. A total of 628 participants (313 women and 315 men, a total of 31 % of the invited sample) provided written informed consent, and they all went through accelerometer registration. Participants with at least 10 h of valid accelerometer data per day for at least four days were included in the data analysis (*n* = 560, 282 women and 278 men) in test phase one. Due to limited capacity at the 10 test centers performing the MSF- and balance testing a total of 30 % of those participating in test phase one were invited to participate in test phase two to assess MSF level and balance. The subjects invited to test phase two were randomly selected and stratified based on sex, age and geographical place of residence. The participants with both valid accelerometer-determined data and MSF- and balance measurements (described below) were included in the final data analysis (*n* = 161, 85 women and 76 men). All the participants in the study provided their written informed consent and they were all informed that they could refuse to participate at any stage in the study.

Approval for the study was granted by the Regional Committee for Medical and Health Research Ethics and the Norwegian Social Science Data Services AS.

### Measurement of musculoskeletal fitness and balance

The MSF- and balance test battery in the present study is partly based on the ALPHA (Assessing Levels of Physical Activity and Fitness) group recommendation by Suni et al. [42], and includes the following tests; one leg standing [43], handgrip strength [44, 45], and static back extension [46]. These established field based tests aiming at adults and older adults, were assigned a score by the ALPHA group [42] from 0 to12 points (where 12 is best) based on the validity, reliability, safety and feasibility. Tests used in the present study were scored as follows: 9 points to the one leg standing test [43], 7 points to the handgrip strength test [44, 45], and 9 points to the static back extension test [46].

The MSF- and balance test battery in the present study also includes tests measuring upper- and lower body flexibility, since the degree of joint flexibility seems to be related to overcome daily life activities (i.e. self-care activities such as feeding ourselves, bathing, dressing, grooming, work, homemaking, and leisure that require physical capability (basic activities of daily living (ADL)) [2]), especially among the older adults [47]. These tests are; sit and reach [48] and back scratch [47]. The sit and reach test has been demonstrated by Lemmink et al. [49] to produce good test-retest reliability in older women and men (intraclass correlations (ICCs): 0.96, 95 % confidence interval (CI): 0.94 to 0.97 and ICCs: 0.98, 95 % CI: 0.97 to 0.99, respectively). The sit and reach test has also been shown to be a valid measure of hamstring flexibility in older women and men (ICCs: 0.57, 95 % CI: 0.39 to 0.71 and ICCs: 0.74, 95 % CI: 0.58 to 0.85, respectively) [50]. The back scratch test has been demonstrated by Rikli and Jones [47] to be a reliable (ICCs: 0.96, 95 % CI: 0.94 to 0.98) and valid (no single criterion available) measure of overall shoulder range of motion (i.e. shoulder joint- and arch flexibility) in older adults.

**Fig. 1:**
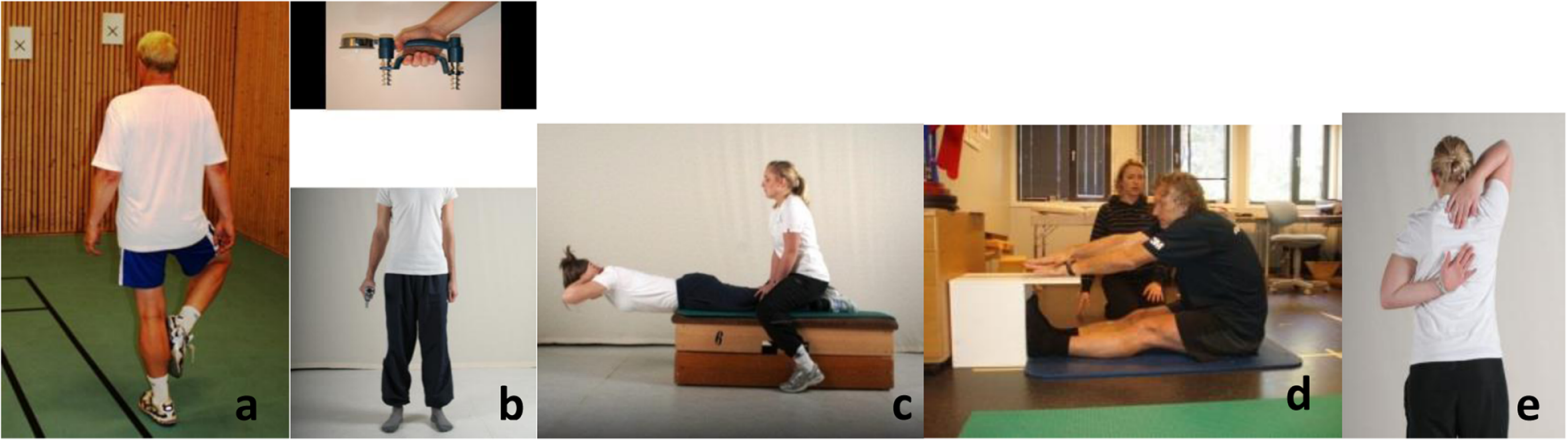
**a**-**e** The musculoskeletal fitness- and balance tests used in the present study. The participants pictured in figure one provided their consent for the publication of this identifiable image

**One leg standing test** [43] measures postural control/static balance. Participants were instructed to stand on their self-selected leg facing a mark at eye height on the wall 3 meters away (Fig. 1a). The free leg heel was placed against the knee joint of the supporting leg with the free-leg externally rotated. The participants’ arms hung alongside their body. One attempt on the self-selected leg was carried out, and the total time the participants managed to keep the initial balancing position was recorded in seconds (sec) (minimum 0 s, maximum 60 s).

**Handgrip strength test** [44, 45] was measured in the dominant hand using a hydraulic dynamometer type baseline 90 kg (kg) (Chattanooga, Hixon, USA, Fig. 1b). The best of three attempts was recorded to the nearest 1 kg.

**Static back extension test** [46] measures endurance capacity of the trunk extensor muscles. Participants were asked to lay face down on a 30 cm tall, 18 cm broad and 135 cm long bench with their iliac crest aligned with the bench’s short side, leaving the upper body beyond the bench and their legs fixed on the bench (Fig. 1c). The participants were instructed to hold their upper body in a horizontal position for as long as they could and the time (in sec) was recorded. Participants were allowed one attempt (minimum 0 s, maximum 240 s).

**Sit and reach test** [48] measures flexibility of the lower back and hamstring musculature. A standardized box (the length of top of the box was 53.3 cm and the height was 32.5 cm) was placed to a wall and the participants sat on the floor with their knees and upper body straight, and their heels against the box. The test was completed with shoes on. The participants reached forward as far as possible along the measuring tape atop of the box, with one hand on top of the other slide along the box and with the back and legs straight (Fig. 1d). The furthest the participants managed to stretch their hands along the measuring tape and hold for two sec, was recorded to the nearest half cm. Point zero, the point where the feet met the box was set at 23 cm from the box’s edge, and the recorded result was 23 cm plus or minus the distance from point zero, depending on what side of point zero the final reach was recorded. One attempt was carried out, and the result was recorded to the nearest half cm.

**Back scratch test** [47] measures flexibility in the shoulder joint and shoulder arch on the right and on the left side. The participants started the test by standing up right, placing one arm/hand on the lower back, moving it up the spine toward their head. The opposite arm/hand was placed behind their neck, moving it down the spine, aiming to place the long finger of each hand as near each other as possible or to overlap the other hand as much as possible (Fig. 1e). The procedure was repeated with opposite arm/hand. The gap between the fingertips of the long finger of both hands was measured to the nearest half cm. The results were recorded to the nearest half cm, as back scratch right arm and left arm over, with positive numbers as long as the fingers overlapped and with negative numbers if the fingers did not meet. One attempt was carried out on each side (right and left arm over), and the result was recorded to the nearest half cm.

### Measurement of physical activity level

ActiGraph GT1M accelerometers (ActiGraph, LLC, Pensacola, FL) were used to quantify the participants’ daily physical activity levels [27, 28]. The accelerometer registers vertical acceleration in units called counts at a rate of 30 Hz in user-defined sampling intervals (epochs) and an embedded pedometer function counts registered the number of steps taken per day [51]. The participants received a pre-programmed accelerometer and written instructions for use by mail. The accelerometer was worn over the right hip for seven consecutive days, but removed while sleeping at night and during water activities such as showering or swimming. After the registration period, the accelerometer was returned using prepaid express mail. The ActiLife software (ActiGraph LLC, Pensacola, Florida, USA) were used to initialize and download the physical activity data and customized SAS based macros (SAS Institute Inc., Cary, NC, USA) were used to derive the physical activity variables. Activity files were deemed valid if a participant accumulated at least 10 h of valid activity recordings per day for at least one day in test phase two. The protocol for collecting the PA data with the Actigraph is in line with the suggestions by Trost et al. [52]. Wear time was defined by subtracting non-wear time from 18 h (all data between 00:00 and 06:00 were excluded) and non-wear time (intervals of at least 60 consecutive minutes with zero counts, with allowance for 1 min with counts >0) were excluded from the analyses.

### Anthropometric variables

Body height and mass were measured to the nearest 0.1 cm and 0.1 kg, respectively, by the use of stadiometers and body mass monitors (Seca opima, Seca, United Kingdom) whilst wearing light clothing and no shoes. Body mass index (BMI) was computed as body mass (kg) divided by meters squared (m^2^).

### Other variables

Chronic diseases, medication for high blood pressure and cardiovascular disease, self-reported health (categorized into: “very good”, “good”, “either good or bad”, “poor/very poor”), and education level (categorized into: < high school, high school, university < 4 years, university ≥ 4 years) were assessed through a questionnaire.

### Statistical analyses

A preliminary Kolmogorov-Smirnov test showed the data to be normally distributed. Data are therefore presented as mean and standard deviations (SD), standard errors (SE), or 95 % confidence interval (CI) when appropriate.

Student’s t-tests for independent samples were used to identify sex differences in continuous variables (age, height, body mass, BMI), and Pearson’s chi-square analyses were used to identify sex differences in categorical variables (chronic diseases, self-reported health, education level) (Table 1).

**Table 1:** Physical cCharacteristics^*a*^ of the study sample

Variable	Women	Men	*p*-value
N	85	76	
^a^Age (yr)	73.2 (5.4)	72.3 (4.8)	0.2
^a^Height (cm)	161.6 (6.0)	175.9 (6.6)	≤0.001
^a^Body mass (kg)	67.0 (10.1)	81.4 (12.2)	≤0.001
^a^BMI (kg/m^2^)	25.7 (3.9)	26.4 (3.0)	0.2
Chronic diseases (%)			
CVD^b^	9.8	16.2	0.2
High BP^c^	30.9	25.3	0.4
Poor mental health	5.9	2.6	0.3
Diabetes type II	4.7	6.5	0.6
Osteoporosis	10.6	2.6	0.04
Rheumatism	24.7	15.5	0.2
COPD^d^	2.4	2.6	0.9
Medication^e^	33.8	41.3	0.3
Self-reported health (%)			
Very good	20.0	21.1	
Good	60.0	63.2	
Either good or bad	16.5	14.5	
Poor/very poor	3.5	1.3	
Education level (%)			
<High school	25.3	26	
High school	43.3	35	
University <4 yr	16.9	23.4	
University ≥4 yr	14.5	15.5	

No significant differences were found in self-reported health and education level between women and men

^a^Data are presented as mean (SD)

^b^Cardiovascular diseases

^c^Blood preassure

^d^Chronic obstructive pulmonary disease

^e^High BP and CVD

Sex and age differences in physical testing results (one leg standing, handgrip strength, static back extension, sit and reach, back scratch right and left arm over) were examined using univariate analysis of variance (Table 2). When examining differences among age groups (65–69 years, 70–74 years, 75–79 years, and 80–85 years), we adjusted for sex and test center, and when examining differences among sexes in the various tests, we adjusted for age and test center. When presenting total values, we adjusted for sex, age, and test center. When we examined differences in MSF- and balance tests in the different age groups the first step was to test the two-way interaction between sex and age groups, by using general linear model. As no significant interaction was found, the analyses were run for both sexes combined.

**Table 2:** Mean (95 % CI) musculoskeletal fitness- and balance test results stratified by age and sex

Variable	65–69		70–74		75–79		80–85		All^a^	
	Women	Men	Women	Men	Women	Men	Women	Men	Women	Men
N	36	36	24	22	15	13	10	5	85	76
OLS (sec)	28.2 (22.3–34.2)	26.3 (20.3–32.3)	15.0 (7.8–22.2)	21.3 (13.7–28.9)	9.2 (0.01–18.3)	10.5 (1.0–20.0)	4.9 (−6.3–16.1)	2.0 (−15.8–19.7)	19.2 (15.4–23.0)	19.8 (15.7–23.8)^a^
*All* ^b^	27.2 (23.1–31.4)^d^		18.0 (12.8–23.2)^e^		9.8 (3.2–16.4)^f^		4.2 (–5.3–13.7)^g^		19.5 (16.7–22.2)^c^	
HG (kg)	27.3 (24.6–30.0)	44.3 (41.6–47.1)	24.2 (20.9–27.5)	40.8 (37.4–44.3)	25.5 (21.3–29.7)	42.7 (38.1–47.2)	21.9 (16.7–27.1)	37.1 (29.8–44.4)	25.6 (23.9–27.4)	42.4 (40.5–44.2)^a,^
*All* ^b^	35.3 (33.4–37.2)		32.0 (29.7–34.4)		33.6 (30.5–36.6)		29.3 (25.1–33.5)		33.5 (32.3–34.8)^c^	
SBE (sec)	73.4 (58.1–88.6)	59.2 (44.6–73.9)	66.5 (48.0–85.0)	54.7 (36.2–73.1)	48.4 (25.7–71.0)	28.6 (5.0–52.2)	49.3 (11.4–87.2)	2.1 (−57.8–62.1)	65.6 (55.6–75.7)	49.6 (39.5–59.7)^a,^
*All* ^b^	66.4 (55.9–76.8)^h^		60.6 (47.7–73.6)		38.6 (22.4–54.8)^i^		32.4 (0.4–64.4)		57.7 (50.6–64.8)^c^	
SR (cm)	23.5 (20.1–26.9)	14.1 (10.7–17.5)	19.2 (15.1–23.3)	14.2 (9.9–18.5)	17.1 (11.9–22.3)	14.3 (8.9–19.7)	15.9 (9.6–22.3)	4.6 (−5.6–14.7)	20.4 (18.2–22.6)	13.4 (11.1–15.8)^a,^
*All* ^b^	18.9 (16.5–21.3)		16.8 (13.8–19.8)		15.8 (12.0–19.5)		11.3 (5.9–16.8)		17.1 (15.5–18.7)^c^	
BSR (cm)	−5.7 (−9.6--1.7)	−12.9 (−16.8- -8.9)	−8.8 (−13.5--4.0)	−12.9 (−18.0- -7.8)	−7.7 (−13.8- -1.7)	−15.8 (−22.1- -9.6)	−13.0 (−20.4- -5.7)	−15.5 (−27.1- -3.8)	−7.7 (−10.2--5.1)	−13.8 (−16.4- -11.1)^a, *^
*All* ^b^	−9.1 (−11.9--6.4)		−10.7 (−14.2--7.3)		−11.6 (−15.9--7.3)		−14.9 (−21.1--8.6)		−10.5 (−12.4--8.7)^c^	
BSL (cm)	−11.2 (−15.2- -7.3)	−19.7 (−23.7- -15.8)	−11.4 (−16.2--6.7)	−17.5 (−22.4- -12.6)	−12.2 (−18.2- -6.2)	−19.3 (−25.5- -13.1)	−18.7 (−26.0- -11.3)	−18.6 (−30.2- -7.0)	−12.3 (−14.8- -9.8)	−19.0 (−21.7- -16.4)^a, *^
*All* ^b^	−15.3 (−18.1--12.6)		−14.3 (−17.7--10.9)		−15.6 (−19.9--11.3)		−20.0 (−26.2--13.8)		−15.5 (−17.3--13.7)^c^	

Abbreviations: *OLS* one leg standing, *HG* handgrip, *SBE* static back extension, *SR* sit and reach, *BSR* back scratch right arm over, *BSL* back scratch left arm over

^*^ *p* < 0.05 between sexes in the different tests

^a^Adjusted for age and test center

^b^Adjusted for sex and test center

^c^Adjusted for age, sex, and test center

^d^65–69 yr compared to 70–74 yr *p* = 0.04, 65–69 yr compared to 75–79 yr *p* ≤ 0.001, and 65–69 yr compared to 80–85 yr *p* ≤ 0.001

^e^70–74 yr compared to 65–69 yr *p* = 0.04

^f^75–79 yr compared to 65–69 yr *p* ≤ 0.001

^g^80–85 yr compared to 65–69 yr *p* ≤ 0.001

^h^65–69 yr compared to 75–79 yr *p* = 0.03

^i^75–79 yr compared to 65–69 yr *p* = 0.03

Linear regression analyses was used to investigate how physical activity level (expressed as 1000 steps increments to aid interpretation of the beta coefficients) was associated with the different MSF- and balance tests (Table 3). The MSF- and balance tests were the dependent variables and 1000 steps increments as the continuous, independent variables. Separate regression models were constructed for each predictor. Crude and adjusted regression coefficients are displayed. Significant interactions between sex*steps and handgrip strength-, sit and reach- and back scratch tests were present. However, running the analyses by sex did not alter any associations in a meaningful way and the analyses are therefore run on the whole sample including age, sex, daily accelerometer wear time and test center as covariates.

**Table 3:** Associations between 1000 steps increments and the different musculoskeletal fitness- and balance variables

	Crude		Adjusted^a^	
	B (SE)	95 % CI	B (SE)	95 % CI
	*OLS (sec)*			
1000 steps increments	2.32 (0.48)^b^	1.36 to 3.28	1.88 (0.52)^b^	0.85 to 2.90
	*HG (kg)*			
1000 steps increments	0.22 (0.32)	−0.41 to 0.84	−1.33 (0.24)	−0.61 to 0.34
	*SBE (sec)*			
1000 steps increments	5.16 (1.21)^b^	2.77 to 7.55	4.63 (1.34)^b^	1.98 to 7.29
	*SR (cm)*			
1000 steps increments	0.44 (0.29)	−0.14 to 1.02	0.15 (0.31)	−0.47 to 0.77
	*BSR (cm)*			
1000 steps increments	0.68 (0.31)^b^	0.06 to 1.29	0.38 (0.35)	−0.31 to 1.067
	*BSL (cm)*			
1000 steps increments	0.76 (0.32)^b^	0.13 to 1.39	0.59 (0.35)	−0.10 to 1.29

Abbreviations: *OLS* one leg standing, *HG* handgrip, *SBE* static back extension, *SR* sit and reach, *BSR*: back scratch right arm over, *BSL* back scratch left arm over

^a^The adjusted models include age, sex, daily accelerometer wear time, and test center as covariates

^b^ *p* < 0.05 between 1000 steps increments and test score

All statistical analyses were conducted using IBM SPSS Statistics 19 for Windows (IBM Corporation, Route, Somers, NY, USA). An α level of *p* ≤ 0.05 was chosen for statistical significance.

## Results

Table 1 shows characteristics of the participants. The mean age (SD) was 73.2 (5.4) years for women and 72.3 (4.8) years for men. Men had significantly greater height and body mass compared to women (*p* ≤ 0.001). No differences were observed between women and men in chronic diseases (except for osteoporosis: 8 % more women reported the disease compared to men, *p* = 0.04), self-reported health, and education level.

### Musculoskeletal fitness and balance by age

Table 2 shows the results from the musculoskeletal fitness- and balance tests, stratified by age and sex. Participants in the youngest age group had significant better results in one leg standing balance compared with the participants in the older age groups; 65–69 years compared with 70–74 years: 9.2 s difference (*p* = 0.04), 65–69 years compared with 75–79 years: 17.4 s difference (*p* ≤ 0.001), and 65–69 years compared with 80–85 years: 23.0 s difference (*p* ≤ 0.001). The youngest age group (65–69 years) had significantly better static back extension endurance compared with the participants aged 75–79 years: 27.8 s difference (*p* = 0.03). We found no statistical age differences in the other musculoskeletal fitness test results.

### Musculoskeletal fitness and balance by sex

The univariate analysis of variance showed that the mean sit and reach results were significantly better in older women (65–85 years) compared with older men (65–85 years) (7.0 cm difference, *p* ≤ 0.001). Both the mean back scratch right- and left arm over results were also significantly better in women compared with men (6.1 cm difference (*p* = 0.01) and 6.7 cm difference (*p* ≤ 0.001), respectively). Also, women had significantly better mean static back extension results compared with men (16.0 s difference, *p* = 0.02). Handgrip strength was significantly better in men compared with women (16.8 kg difference, *p* ≤ 0.001). We found no significant sex differences in mean one leg standing balance time.

### Physical activity levels, musculoskeletal fitness and balance

Table 3 shows the associations between 1000 steps increments and the different musculoskeletal fitness- and balance tests. Linear regression analyses showed that increased daily step counts were associated with significantly better test scores for the one leg standing test and the static back extension test in older adults (65–85 years). For the one leg standing test, an increase of 1000 steps per day was associated with approximately 2 s better performance on the test (b = 1.88, 95 % CI: 0.85 to 2.90, *p* ≤ 0.001), equivalent to 9.6 %. For the static back extension test, an increase of 1000 steps per day was associated with approximately 5 s better performance on the test (b = 4.63, 95 % CI: 1.98 to 7.29, *p* = 0.001), equivalent to 8.9 %. For the hand grip test, an increase of 1000 steps per day was associated with approximately −1.3 kg in performance on the test (b = −1.33, 95 % CI: −0.61 to 0.34, *p* = 0.6). For the sit and reach test, an increase of 1000 steps per day was associated with approximately 0.2 cm in performance on the test (b = 0.15, 95 % CI: −0.47 to 0.77, *p* = 0.6). For the back scratch test, right and left arm over, an increase in 1000 steps per day was associated with approximately 0.4 cm (b = 0.38, 95 % CI: −0.31 to 1.07, *p* = 0.3) and 0.6 cm (b = 0.59, 95 % CI: −0.10 to 1.29, *p* = 0.09), respectively.

## Discussion

The aims of the present study were to; 1) describe musculoskeletal fitness and balance in a random national sample of Norwegian older individuals (65–85 years); 2) examine age- and sex-related differences in musculoskeletal fitness and balance, and 3) to investigate the association among musculoskeletal fitness, balance, and objectively-assessed physical activity levels. The main findings were that the youngest participants (65–69 years) had significantly better static balance and muscular endurance in the trunk extensors compared with the older participants. Also, Norwegian older women (65–85 years) had significantly better upper and lower body flexibility, in addition to better muscular endurance in the trunk extensors compared with older men (65–85 years), whereas the Norwegian older men (65–85 years) had significantly better handgrip strength compared with older women (65–85 years). No sex differences were found in static balance. Further, a daily increment of 1000 steps was associated with significantly better static balance and muscular endurance in trunk extensors in older individuals (65–85 years).

We found significantly better static balance and muscular endurance in the trunk extensors among the youngest participants (65–69 years) compared with the older participants. Similar results have been found in one other study [15]. This finding might be connected to differences in physical activity level across age groups. We have previously shown a 50 % higher physical activity level among the youngest participants (65–70 years) compared with the oldest participants (80–85 years) [28]. Another possible explanation might be that increasing age leads to a progressive loss of balance [2–4] and muscular strength and endurance [1], mostly because of degenerative processes in the central and peripheral nervous system [53] and qualitative and quantitative changes in the muscular system [3]. For joint flexibility and handgrip strength we found no significant differences between the youngest and the older age groups, differences which have been observed in other studies [16, 17, 21, 22]. This discrepancy might be a result of differences in socioeconomic status, cultural differences with respect to retirement age, infrastructure and degree of environmental security among the populations studied.

We found significantly better joint flexibility in older women (65–85 years) than in older men (65–85 years) which is in accordance with findings from previous studies [21, 22, 37, 47, 54]. A possible explanation for these sex-related differences in joint flexibility might be related to differences in physical activity patterns among older men and women. We have previously shown that Norwegian older women spent more time (minutes) on low-intensity physical activity than did their male counterparts [28]. This observation was confirmed in the present study because we found that women spent significantly more time each day performing low-intensity physical activity compared with the men (216 versus 190 min (*p* = 0.001), respectively) (data not shown). We could therefore speculate whether daily low-intensity activities such as washing dishes, hanging washing, ironing and cooking might affect joint flexibility in older women by limiting the age- and activity-related deterioration. Other factors that might play a role regarding sex-related differences in joint flexibility are: anatomical and physiological differences, smaller muscle mass and different joint geometry and collagenous muscle structure [55]. Older Norwegian men and women also seemed to have somewhat better mean flexibility in lower back and hamstring musculature than what has been reported among elderly in the USA [47] and among elderly in Spain [21]. This discrepancy might be explained by different test procedures as the two latter studies used chair sit and reach test, in addition to including a broader age range (60–85+). Shoulder joint- and arch flexibility also seemed to be somewhat better among older Norwegian men and women compared with older men and women in Spain [21]. The exact same test procedure was used in the two studies. Therefore, the discrepancy might be related to differences in sample sizes and age ranges as Gusi et al. [21] included 6.449 participants aged 60–94 years old.

Furthermore, we also found significantly better muscular endurance in the trunk extensors in women than in men. This sex-related difference might be related to biomechanical load differences during the static back extension testing, meaning that women’s shorter and lighter upper body compared with the longer and heavier upper body of men creates a shorter resistance arm resulting in relatively lower torque demands to maintain back extension in women than in men. This may make it easier for women to maintain the correct position for a longer period. In addition, women might be performing more domestic activities on a daily basis than men which require them to stand in an upright position (e.g. when washing dishes, hanging washing, ironing, and cooking) [56]. This might affect the muscular endurance capacity in the trunk extensors by limiting age- and activity-related deterioration [57].

Men had significantly better handgrip strength than women, which is in accordance with other cross-sectional studies where dynamometers were used [16–19, 21]. Our population appeared to have somewhat better handgrip strength than what has been reported in studies from Brazil and Australia [18, 19]. This discrepancy might be related to different selection of participants, cultural differences with respect to sex equality across countries (e.g. distribution of work regarding household and gardening), in addition to differences in test procedure, like measuring grip strength seated [19] instead of standing in an up-right position which was done in the present study. It has to be mentioned though, that this comparison is based on a difference in age range (65–85 years versus ≥70 years), which also has to be taken into consideration when comparing our findings with the referred studies above.

We found no sex differences in static balance which is in contrast to one other study, showing significantly better static balance in older men than in older women [54]. A possible explanation for not finding any sex-related difference in the static balance among older Norwegian adults might be related to their physical activity level. We have previously reported no sex-related differences in overall physical activity level within the different age groups among older Norwegian adults [28]. This observation was confirmed in the present study, as we found no sex-related differences in the number of steps taken per day (7551 for women versus 7356 for men, *p* = 0.7) (data not shown). Norwegian older men and women seemed to have better static balance compared with 60–80 year old Iranian men (*n* = 36) and women (*n* = 40) [54]. Older Norwegian women appeared to have somewhat lower static balance results compared with what has been reported among 60–86 year old American women (*n* = 71) [15]. This variation in measured values for one leg standing time might be related to differences in the populations examined (e.g. sample size, high versus low functioning elderly) as well as procedural differences (e.g. shoes on, barefooted, dominant-, non-dominant leg, eyes open, eyes closed), which might affect the results [58].

We found that a daily increment of 1000 steps was associated with significantly better static balance and muscular endurance in the trunk extensors in older Norwegian individuals. This knowledge may be of importance towards developing and initiating future preventive health strategies aiming at older adults. Attention should be given to balance and muscular endurance, as both components seem to have relevance to overcome activities of daily living [8, 57]. A recently published study by de Melo et al. [34] reported that agility/balance was significantly associated with pedometer-assessed steps taken per day when comparing older Canadian adults categorized as “high walkers” (mean steps for 3 days: ≥6500) with “low walkers” (mean steps for 3 days: <3000) (*n* = 60, mean age 76.9 years). However, body sway/static balance was unrelated to accelerometer-defined measurement, expressed as daily step counts, in older Japanese men (*n* = 94) and women (*n* = 76), aged 65–84 years [38]. In addition, hand grip strength was also unrelated to daily step counts in this elderly Japanese cohort, which is in line with our results. Furthermore, we found no association between a daily increase of 1000 steps and upper- and lower joint flexibility. In contrast, de Melo et al. [34] reported significantly better lower body flexibility in “high walkers” than in “low walkers”. To our knowledge, no prior work has examined the associations between muscular endurance in the trunk extensors and physical activity among older adults, which makes our results rather novel. However, there are existing studies [59–61] looking at the association between muscular endurance in the trunk extensors, physical activity and health related factors. These studies are all aiming at younger age groups, in addition to use of subjectively-assessed physical activity level through a questionnaire, which makes a comparison rather inappropriate.

One of the major strength of this study is the use of standardized musculoskeletal fitness and balance tests, with high validity, reliability, safety and feasibility. Furthermore, we used an objective assessment of physical activity, and the participants showed good compliance with the protocol and few data were lost because of insufficient wearing time or defect monitors. The participants achieved a mean of 6.6 days (SD 1.4) with valid activity recordings, and the mean wear time was 14.0 h per day (SD 1.2) [28].

We acknowledge some limitations to our study. The relatively low participation rate might question the representativeness of the data. A drop-out analysis performed via registry linkage showed that the responses varied according to socio-demographic variables [27]. Several test centers and test leaders were involved in the data collection and this might have influenced the reliability of the data. To minimize this limitation a test protocol together with illustrating test procedure posters were developed, followed by a pilot study where all the tests were accomplished prior to the main study. Also, the test leaders were trained in the test protocol and test procedures. Furthermore, there are limitations worth noting when interpreting accelerometry data [62]. Walking technique must be taken into consideration because it can affect the validity of accelerometer step counts, especially in older individuals [62]. It appears that some accelerometers can undercount activity in individuals with a nonstandard gait (e.g. upper body angled forward and knees bent during walking), thereby underestimating the activity level in these individuals [63]. Another limitation is that only one test of static balance was included and that muscular strength was only examined via handgrip dynamometer. Also, as in any observational study, we have to be cautious in inferring causality based on our findings.

## Conclusion

The youngest participants (65–69 years) among older Norwegians had significantly better static balance and muscular endurance in trunk extensors compared with the older participants. Older Norwegian women (65–85 years) had significantly better upper and lower body flexibility, in addition to significantly better muscular endurance in the trunk extensors compared with older men (65–85 years), whereas older Norwegian men (65–85 years) had significantly better hand grip strength compared with older women (65–85 years). No sex differences were found in static balance. A higher physical activity level, expressed as daily increments of 1000 steps, was associated with significantly better static balance and muscular endurance in the trunk extensors in older Norwegians (65–85 years). This study provides important normative data on commonly used physical fitness tests in older Norwegians. Further investigation of trunk endurance and static balance as key foci for interventions to increase physical activity in older men and women is warranted.

## Abbreviations

BSL: Back scratch left arm over
BSR: Back scratch right arm over
CI: Confidence interval
HG: Hand grip
MSF: Musculoskeletal fitness
OLS: One leg standing
PA: Physical activity
SD: Standard deviation
SR: Sit and reach
